# Wnts in action: from synapse formation to synaptic maintenance

**DOI:** 10.3389/fncel.2013.00162

**Published:** 2013-11-05

**Authors:** Ellen M. Dickins, Patricia C. Salinas

**Affiliations:** Department of Cell and Developmental Biology, University College LondonLondon, UK

**Keywords:** synaptogenesis, synapse disassembly, synaptic plasticity, Frizzled, Dvl, LTP, neurodegenerative disease

## Abstract

A proper balance between synapse assembly and disassembly is crucial for the formation of functional neuronal circuits and synaptic plasticity in the adult brain. During development, synaptogenesis generates a vast excess of synapses, which are subsequently eliminated. Importantly, aberrant synaptic disassembly during development underpins many neurological disorders. Wnt secreted proteins are robust synaptogenic factors that regulate synapse assembly and function in the developing and mature brain. Recent studies show that Wnt blockade with the antagonist Dickkopf-1 (Dkk1) induces the rapid disassembly of synapses in mature neurons. Importantly, Dkk1 mediates synaptic loss induced by Amyloid-ß, a key pathogenic molecule in Alzheimer’s disease (AD). These findings provide new insights into the potential contribution of dysfunctional Wnt signaling to synaptic loss observed in neurodegenerative diseases. In this review, we discuss the role of Wnt signaling in vertebrate synaptic assembly, function and maintenance, and consider how dysfunction of Wnt signaling could contribute to synaptic disassembly in neurodegenerative diseases such as AD.

## Introduction

During early development, Wnts regulate critical cellular processes such as cell proliferation and cell fate, neuronal polarity and migration. In addition, Wnts modulate dendritogenesis, axon guidance and synaptogenesis (Ciani and Salinas, 2005; Ille and Sommer, 2005; Rosso et al., 2005; Salinas and Zou, 2008; Budnik and Salinas, 2011; Park and Shen, 2012; Salinas, 2012). The array of diverse cellular processes regulated by Wnt signaling is achieved through multiple Wnt ligands interacting with numerous receptors and co-receptors that trigger distinct signaling cascades that induce local changes and/or global changes through the modulation of target gene expression (Mikels and Nusse, 2006; Kikuchi et al., 2007; van Amerongen and Nusse, 2009). Additional levels of complexity are conferred by the temporal and spatial expression of secreted regulatory factors, which antagonize or activate specific Wnt pathways, or act as a switch between different Wnt pathways. Several excellent reviews on Wnt signaling pathways and their cellular outcomes are available (Logan and Nusse, 2004; Kohn and Moon, 2005; Gordon and Nusse, 2006; Kikuchi et al., 2007; Angers and Moon, 2009; van Amerongen and Nusse, 2009). Therefore, the different Wnt pathways will not be discussed here.

**Figure 1:**
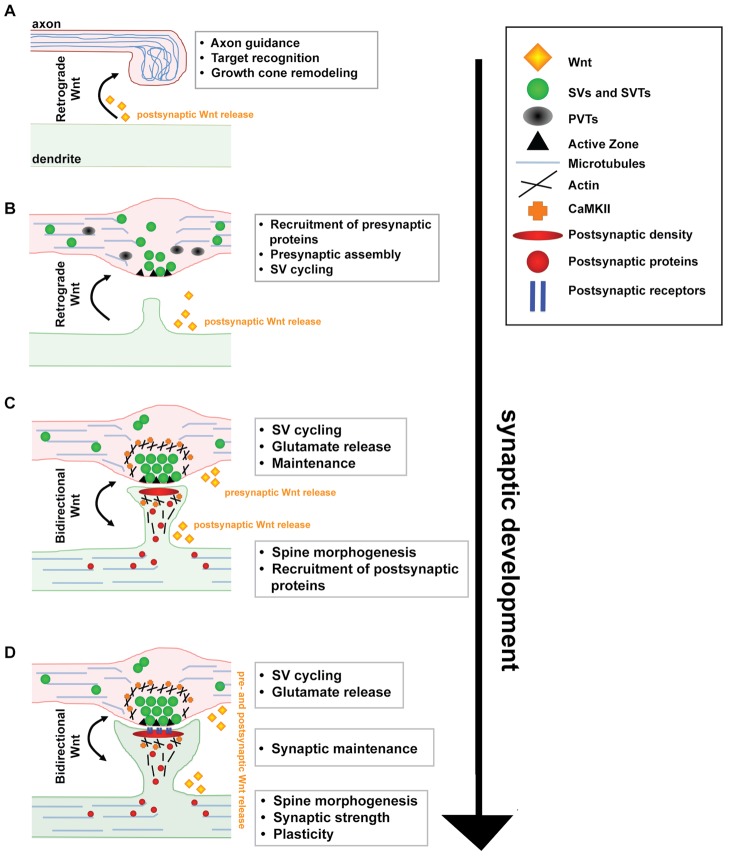
**A model for the function of Wnt signaling during synapse development and maintenance**. **(A)** Target derived Wnt signals guide incoming axons and induce axon and growth cone remodeling by modulating the cytoskeleton. **(B)** Wnt signals directly to the axon through a divergent canonical pathway to stimulate the recruitment of presynaptic proteins resulting in the formation of synaptic boutons. **(C)** Wnt also signals directly to the postsynaptic dendrite through CaMKII to stimulate spine morphogenesis, postsynaptic protein recruitment and synaptic strength. **(D)** At mature synapses, Wnt signaling regulates synaptic function and maintenance.

Studies in the past decade have demonstrated that Wnts are key synaptic organizers that play a critical developmental role in establishing neural circuits. The formation of functional synapses requires the precise assembly of pre- and postsynaptic sites in perfect apposition. This process ultimately depends upon the molecular dialogue between the pre- and postsynaptic sides (Figure 1). Indeed, Wnt factors are key players that signal to both pre- and/or postsynaptic sites to promote synapse assembly, morphology and function (Hall et al., 2000; Krylova et al., 2002; Ahmad-Annuar et al., 2006; Cerpa et al., 2008; Davis et al., 2008; Henriquez et al., 2008; Farias et al., 2009; Gogolla et al., 2009; Cuitino et al., 2010; Varela-Nallar et al., 2010; Ciani et al., 2011). Wnts, their cognate receptors and several signaling components continue to be expressed in the adult brain, suggesting a role for Wnts in the mature nervous system. Whilst the function of Wnt signaling in the adult is less understood, recent studies indicate a role in synapse maintenance and plasticity (Chen et al., 2006; Gogolla et al., 2009; Cerpa et al., 2011; Ciani et al., 2011; Purro et al., 2012).

Here we discuss the role of Wnt signaling in synapse formation and maintenance. We will first review how Wnts contribute to synapse assembly and function, focusing on the mammalian central nervous system. This prologue is important to highlight the importance of Wnt signaling at the synapse and the consequence of Wnt blockade or Wnt dysfunction on synapse instability observed in certain neurodegenerative diseases.

### Presynaptic remodeling

As axons enter into their target field, they undergo extensive modeling. Studies in cerebellar mossy fiber axons and dorsal root ganglion cells (DRGs) reveal that Wnt signaling activates a divergent-canonical pathway through Disheveled (Dvl) resulting in the inhibition of Glycogen synthase kinase-3 ß (Gsk3ß) to regulate axonal remodeling (Lucas and Salinas, 1997; Lucas et al., 1998; Krylova et al., 2002; Purro et al., 2008). This pathway is independent of transcription and induces profound changes in growth cone size and axonal microtubule dynamics by affecting Gsk3β-mediated phosphorylation of microtubule-associated proteins, such as MAP1B, and the localization of Adenomatous polyposis coli (APC) (Ciani et al., 2004; Salinas, 2007; Purro et al., 2008). APC, in addition to being a component of the canonical-Wnt signaling destruction complex, is a microtubule plus-end binding protein that captures the distal end of microtubules to the leading edge of the growth cone (Galjart, 2005). During axon remodeling, Wnt3/3a signals through Dvl1 to inhibit Gsk3β resulting in the loss of APC from microtubule plus ends. Wnt-induced APC loss from microtubules results in the loss of directionality of microtubule growth and the subsequent formation of lopped microtubule within growth cones (Purro et al., 2008). These looped microtubules provide a structural mechanism for capturing molecules required for presynaptic differentiation and the transformation of motile growth cones into presynaptic boutons. Further studies are required to determine how microtubule dynamics and their organization contribute to early stages of presynaptic differentiation and whether Wnts regulate microtubule dynamics, not only at growth cones, but also at the axon shaft to promote the formation of *en passant* synapses.

### Presynaptic assembly

**Figure 2:**
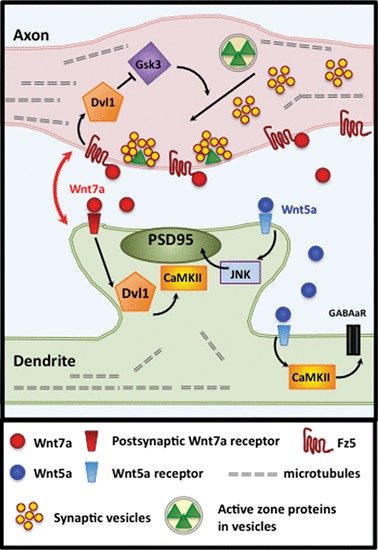
**Wnt7a signals bi-directionally at vertebrate synapses.** At hippocampal synapses, coordinated bidirectional Wnt7a signaling regulates synapse assembly, development and maintenance. In the axon, Wnt7a binds with Fz5 to activate a divergent-canonical pathway through Dvl1 and Gsk3β that promotes the recruitment of synaptic components, including SV and active zone proteins. In postsynaptic dendrites, Wnt7a promotes spine morphogenesis, PSD-95 recruitment and synaptic strength by signaling via Dvl1 and CaMKII. The postsynaptic Wnt receptor has yet to be determined. Wnt5a signals postsynaptically to promote PSD95 clustering via JNK activation and GABA_A_ receptor clustering via CaMKII. The postsynaptic Wnt5a receptor has yet to be identified.

Several Wnts promote the assembly of presynaptic release sites. Wnt7a/b and Wnt3/3a stimulate the accumulation of a number of functionally diverse presynaptic proteins including Synapsin 1, vGlut1 and Bassoon, as well as synaptic vesicles (SVs) (Hall et al., 2000; Ahmad-Annuar et al., 2006; Cerpa et al., 2008; Ciani et al., 2011; Figure 2). In contrast, blockade of Wnt signaling by secreted frizzled-related proteins (Sfrps) or Dickkofp-1 (Dkk1) inhibits the ability of Wnt7a/b to induce presynaptic protein clustering (Ahmad-Annuar et al., 2006; Davis et al., 2008). Critically, mice deficient in Wnt7a and/or Dvl1 display significant defects in presynaptic protein clustering (Hall et al., 2000; Ahmad-Annuar et al., 2006). Time-course analyses reveal that Wnt7a/b induces synaptic protein clustering within 15 min demonstrating a rapid synaptogenic effect (Ahmad-Annuar et al., 2006; Ciani et al., 2011). Furthermore, Wnts rapidly increase the number of functionally active presynaptic sites without affecting total levels of synaptic proteins (Ahmad-Annuar et al., 2006; Cerpa et al., 2008; Varela-Nallar et al., 2009). These findings suggest that Wnts induce presynaptic assembly by promoting the recruitment of existing synaptic proteins and SVs.

Signaling through the seven transmembrane Frizzled (Fz) receptors promotes presynaptic organization. Wnt3a and Wnt7a bind to the Fz1 and Fz5 receptors, respectively, to stimulate presynaptic assembly (Varela-Nallar et al., 2009; Sahores et al., 2010). Both receptors localize at synapses and stimulate the clustering of active zone components and SVs. Moreover, loss of Fz1 or Fz5 function by using the respective soluble cysteine-reach domains (CRD) or shRNA-mediated knockdown of Fz5 blocks Wnt-mediated presynaptic assembly (Varela-Nallar et al., 2009; Sahores et al., 2010). In addition to Fz receptors, Wnts can regulate presynaptic assembly by binding to other receptors. For example, Wnt5a binds to Ror tyrosine kinase receptors to stimulate Synaptophysin clustering in cultured hippocampal neurons (Paganoni et al., 2010). Further studies are required to determine whether different Wnt isoforms selectively bind with specific receptors to regulate distinct aspects during synapse assembly.

Wnt signals via a divergent canonical pathway that is independent of transcription, to stimulate presynaptic assembly. A number of findings support this conclusion. Firstly, blockade of canonical-Wnt signaling at the receptor level by the secreted antagonist Dkk1 prevents Wnt-induced presynaptic differentiation (Davis et al., 2008). Secondly, expression of Dvl1, which localizes to presynaptic sites, is sufficient to induce clustering of presynaptic proteins and to promote the formation of functional neurotransmitter release sites (Ahmad-Annuar et al., 2006). In contrast, neurons from *Dv11* null mice exhibit fewer neurotransmitter release sites and respond poorly to exogenous Wnt7a/b (Ahmad-Annuar et al., 2006). In the canonical-Wnt pathway, activation of Dvl1 inhibits the serine/threonine kinase Gsk3β, which phosphorylates and targets β-catenin for degradation. Gsk3β is expressed presynaptically and its pharmacological inhibition mimics Wnt-induced clustering of synaptic components (Lucas and Salinas, 1997; Hall et al., 2000; Ahmad-Annuar et al., 2006; Davis et al., 2008). Together these findings support a role for the canonical Wnt signaling pathway in presynaptic assembly. However, blockade of transcription by RNA polymerase inhibition does not affect Wnt mediated presynaptic assembly (EM Dickins and PC Salinas, unpublished results) neither axonal remodeling, a process that precedes presynaptic assembly (Purro et al., 2008). These results suggest that Wnt might signal locally to regulate Dvl1 and Gsk3β to promote presynaptic assembly.

How does Wnt signaling promote the assembly of synaptic boutons? Previous studies have shown that a divergent-canonical Wnt pathway regulates microtubule dynamics in the axon shaft and the growth cone to induce axonal branching, growth cone spreading, and changes in bouton morphology; such effects depend upon profound changes in microtubule organization (Lucas and Salinas, 1997; Hall et al., 2000; Purro et al., 2008). Thus, local regulation of microtubule dynamics by Wnts could provide a possible mechanism for directed delivery of synaptic components to future synaptic sites. While this model is consistent with the changes observed in the formation of terminal boutons, most synapses in the central nervous system are *en passant*. Therefore, it remains to be determined how *en passant* boutons become assembled. Interestingly, Wnts seem to promote microtubule unbundling along the axon shaft in some neurons suggesting that changes in microtubule organization might contribute to the initial recruitment of synaptic components to future synaptic boutons. Further studies are required to determine the mechanisms by which Wnt signaling stimulates the rapid recruitment of presynaptic proteins.

### Postsynaptic assembly

Wnts also signal to dendrites to promote the recruitment of postsynaptic components (Cerpa et al., 2008; Henriquez et al., 2008; Farias et al., 2009; Cuitino et al., 2010; Ciani et al., 2011; Jensen et al., 2012; Figures 1, 2). Interestingly, different Wnt isoforms specifically regulate the assembly of excitatory and/or inhibitory synapses. Wnt7a exclusively stimulates the formation of excitatory synapses, without affecting inhibitory synapses (Ciani et al., 2011). Wnt7a stimulates PSD95 recruitment and the apposition of excitatory pre- and postsynaptic markers (Ciani et al., 2011). Wnt7a promotes excitatory synapse formation by inducing the formation and growth of dendritic spines, structures that primarily receive excitatory inputs. Conversely, *Wnt7a; Dvl1* mutant mice exhibit significant deficits in spine number and morphology in the Cornus Ammonis (CA) CA1 and CA3 regions of the hippocampus (Ciani et al., 2011). These mice also exhibit reduced frequency and amplitude of alpha amino-3-hydroxyl-5-methyl-4-isoxazolepropionic acid receptor (AMPAR)-mediated miniature excitatory postsynaptic currents (mEPSCs). Although the mechanism by which Wnt7a increases spine number remains to be elucidated, spine growth is induced through Dvl1 and local activation of CaMKII at dendritic spines (Ciani et al., 2011).

In contrast to Wnt7a, Wnt5a acts as a pan-synaptogenic factor that stimulates PSD95 clustering at excitatory synapses via JNK activation (Farias et al., 2009) and *γ*-aminobutyric acid type A (GABA_A_) receptor clustering at inhibitory synapses through CaMKII signaling (Cuitino et al., 2010; Figure 2). Whilst some of the signaling mechanisms by which Wnts promote postsynaptic assembly have been identified, many questions remain unanswered. For instance, the identity of the postsynaptic receptors for Wnts and the mechanisms that regulate the formation of different types of synapses are currently unknown.

The precise role of Wnt5a in synaptic assembly remains poorly understood. Wnt5a has been reported to stimulate spine formation via calcium signaling in cultured hippocampal neurons (Varela-Nallar et al., 2010). However, these results have not been consistently recapitulated in brain slices (Cerpa et al., 2011), and another group reported that Wnt5a inhibits excitatory presynaptic assembly (Davis et al., 2008). The reason for these apparent opposing results is unclear at present. Possible explanations could be the age and/or type of neuronal preparation studied, different sources of Wnt5a (recombinant versus conditioned media from Wnt5a-expressing cells), the concentration used or the exposure time. Further work is required to gain insight into the function of Wnt5a as a positive or negative regulator of excitatory synaptogenesis, and how Wnt5a stimulates postsynaptic differentiation of both excitatory and inhibitory synapses, whereas Wnt7a only promotes excitatory synapse formation.

### Bidirectional Wnt signaling

A number of studies have shown that Wnts can signal in an anterograde and retrograde manner. For example, at the neuromuscular junction (NMJ), Wnt release from motoneurons regulates postsynaptic differentiation on muscle cells (Krylova et al., 2002; Packard et al., 2002; Henriquez et al., 2008; Jensen et al., 2012). In contrast, in the cerebellum, Wnt7a/b is released from postsynaptic cells to regulate presynaptic assembly and remodeling (Hall et al., 2000). In the hippocampus, several Wnts are expressed but the exact source of Wnts at the mossy fiber-granule cell (MF-GC) and Cornus Ammonis (CA3-CA1) synapse remains poorly understood. Recent studies suggest that Wnt7a/b protein is present in the Dentate Gyrus (DG), CA3 and CA1 neurons (Gogolla et al., 2009; Ciani et al., 2011). However, where and how Wnts are secreted to regulate synapse formation and function remains to be elucidated.

Coordinated bidirectional signaling contributes to Wnt mediated synaptic assembly. At the *Drosophila* NMJ, the Wnt family member Wingless (Wg) signals to both sides of the synapse (Packard et al., 2002; Ataman et al., 2008). Similarly, at hippocampal synapses, Wnt7a acts bidirectionally on axons and dendrites suggesting a conserved role for bidirectional Wnt signaling in synapse assembly between vertebrates and invertebrates. However, pre- and postsynaptic assembly is not concurrent. Time-course analysis shows that Wnt7a induces clustering of the presynaptic protein vesicular glutamate transporter1 (vGlut1) within 15 min, whereas clustering of the postsynaptic scaffold protein PSD95 takes longer (Ciani et al., 2011). These results suggest that the presynaptic terminal responds faster to Wnt7a than the postsynaptic side. It is currently unclear whether Wnt7a acts through different receptors to trigger different signaling cascades to coordinate the assembly at both sides of the synapse.

### Synaptic function

The initial stages of synaptic differentiation occur within the first few hours after the establishment of the axo-dendritic contact. However, the development of a nascent synapse into a functional synapse involves the recruitment of hundreds of proteins, morphological changes and establishment of functional electrophysiological properties (Zhang and Benson, 2001; Knott et al., 2006; Nagerl et al., 2007).

Wnt signaling participates in presynaptic function by promoting the formation of more SV recycling sites and increasing neurotransmitter release (Ahmad-Annuar et al., 2006; Cerpa et al., 2008; Varela-Nallar et al., 2009). In hippocampal neurons, the intracellular activation of the canonical-Wnt signaling by expression of Wnt signaling components or by bath application of Wnt3a or Wnt7a increases the frequency of spontaneous and mEPSCs by a divergent-canonical pathway that mobilizes calcium and is independent of transcription (Beaumont et al., 2007; Cerpa et al., 2008; Avila et al., 2010). Further analyses of SV cycling dynamics showed that Wnt7a specifically enhances SV exocytosis to facilitate neurotransmitter release (Cerpa et al., 2008). Importantly, electrophysiological studies in the cerebellum of *Wnt7a; Dvl1* mutant mice reveal defects in the frequency of mEPSCs, without apparent changes at the structure of active zones as determined by electron microscopy (Ahmad-Annuar et al., 2006). These results suggest that Wnt signaling may be required for neurotransmitter release. Consistent with this hypothesis, Dvl1 has been shown to bind directly to Synaptotagmin (Kishida et al., 2007), a key protein in neurotransmission. Dvl1 appears to regulate SV exo- and endocytosis in PC12 cells (Kishida et al., 2007). Together these studies demonstrate that Wnt signaling contributes to the assembly of functionally active presynaptic sites. However, the role of Wnt signaling in neurotransmitter release remains to be fully demonstrated.

On dendrites, Wnt7a increases the growth and maturation of spines manifested by increased size and PSD95 content (Ciani et al., 2011). In hippocampal neurons, Wnt7a increases spine size by almost 50% within 3 hrs, and by 65% within 16 hrs indicating a rapid and progressive spine growth. Consistent with an increased spine head size, Wnt7a signaling regulates synaptic strength as determined by defects in evoked excitatory postsynaptic currents (EPSCs) in *Wnt7a; Dvl1* mutant mice. Postsynaptic activation of the Wnt pathway by expression of Dvl1 also stimulates spine growth, increases the amount of PSD95 within dendritic spines and the number of spines containing PSD95 (Ciani et al., 2011). Several findings demonstrate that Wnt7a through Dvl1 and CaMKII modulates spine growth and synaptic strength by rapidly activating CaMKII within dendritic spines (Ciani et al., 2011; Figure 2). Given the role of CaMKII in the structural and functional plasticity of synapses, these findings raise the interesting possibility that Wnt7a signaling participates in postsynaptic plasticity in the adult brain.

Wnt signaling also modulates inhibitory synapse formation and function (Figure 2). Wnt5a increases the insertion and clustering of GABA_A_ receptors in cultured hippocampal neurons (Cuitino et al., 2010). Moreover, evoked recordings demonstrate that Wnt5a rapidly increases the amplitude of GABA-mediated currents without affecting the pair pulse index suggesting the Wnt5a might act postsynaptically. This postsynaptic effect is blocked by KN93 suggesting that Ca2+/calmodulin dependent kinase (CaMKs) are involved (Cuitino et al., 2010). Although it remains to be determined whether Wnt5a is necessary for the postsynaptic function of inhibitory synapses, these results demonstrate that different Wnts can promote excitatory and/or inhibitory synapse function.

### Neuronal activity regulates Wnt levels and Frizzled receptor trafficking

A number of studies have shown that neuronal activity stimulates the expression and/or release of Wnts in the developing and mature nervous system (Yu and Malenka, 2003; Chen et al., 2006; Wayman et al., 2006; Gogolla et al., 2009; Sahores et al., 2010). Depolarization of young hippocampal neurons stimulates Wnt release, which in turn regulates dendritogenesis (Yu and Malenka, 2003; Wayman et al., 2006). In cultured hippocampal neurons, activation of N-methyl-D-aspartate receptors (NMDARs) through tetanic stimulation induces Wnt3a release resulting in the activation of the canonical Wnt pathway (Chen et al., 2006). Wnt2 is also regulated by activity through a mechanism that requires NMDAR activation and cAMP response element-binding protein (CREB)-mediated transcription to promote dendritic arborization of cultured hippocampal neurons (Wayman et al., 2006). Moreover, *in vivo* studies show that environmental enrichment (EE) stimulates Wnt7a/b expression in the adult mouse hippocampus during the remodeling of mossy fiber terminals that contact CA3 dendrites. Importantly, blockade of endogenous Wnts by local microinjection of the Wnt antagonist Sfrp1 suppresses activity-dependent presynaptic remodeling and synaptogenesis in the CA3 region (Gogolla et al., 2009). Collectively, these studies demonstrate that neuronal activity promotes the expression and/or release of Wnt ligands to regulate dendritic development and adult synaptic remodeling.

**Figure 3:**
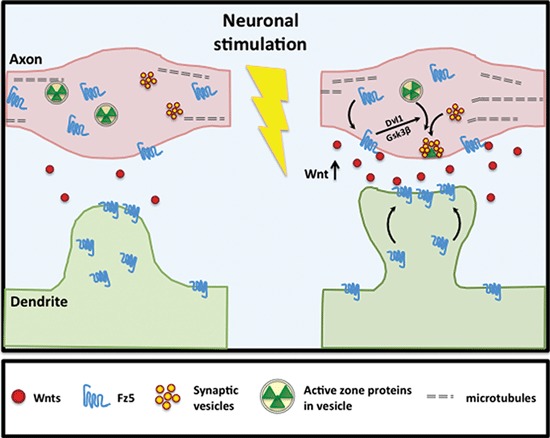
**Neuronal activity stimulates Fz5 mobilization to synapse cell surface.** Neuronal activity stimulates Wnt expression and/or release. Activity-dependent Wnt release subsequently induces Fz5 cell surface expression at synaptic sites. In hippocampal neurons, activity-dependent Fz5 mobilization through Wnt signaling is required for activity-mediated synapse formation.

Activity-dependent Wnt release induces Fz receptor surface localization, which in turn promotes synapse assembly (Figure 3). In cultured hippocampal neurons, Fz5, a receptor for Wnt7a, translocates to the cell surface and to synaptic sites following high frequency stimulation (HFS; Sahores et al., 2010), a protocol that induces long-term potentiation (LTP). Importantly, the Wnt antagonists Sfrps or the CRD of Fz5 that sequesters endogenous Wnts that bind to the Fz5 receptor, block the synaptic localization of Fz5 during HFS. These findings suggest that neuronal activity promotes the expression and/or release of Wnt factors, which are required for Fz5 trafficking to the cell surface and to the synapse. Importantly, Wnt blockade also prevents HFS-mediated synapse assembly (Sahores et al., 2010). These findings demonstrate that Wnt-Fz5 signaling is required for activity-mediated synapse formation. It remains to be identified which Wnt isoforms are modulated by activity to promote Fz5 trafficking to the synapse.

### Wnt signaling and functional plasticity of mature synapses

Synaptic plasticity refers to enduring changes in synaptic strength in response to different patterns of neuronal activity. Long-term changes to presynaptic neurotransmitter release and/or postsynaptic signal transduction through receptor availability and downstream effectors mediate synaptic plasticity. Synaptic plasticity mediates changes in sensory, motor and somatosensory inputs, as well as learning and memory. The patterns of expression of Wnts strongly suggest that they regulate these processes in the brain.

Wnts are released by activity-dependent mechanisms (Yu and Malenka, 2003; Chen et al., 2006; Wayman et al., 2006; Gogolla et al., 2009). Furthermore, Wnts and many of their signaling components continue to be expressed in the adult brain (Coyle-Rink et al., 2002; Shimogori et al., 2004; De Ferrari et al., 2007; Gogolla et al., 2009). Increasing evidence now suggests that sustained Wnt signaling may contribute to synaptic plasticity (Chen et al., 2006; Beaumont et al., 2007; Ataman et al., 2008; Cerpa et al., 2008; Gogolla et al., 2009; Varela-Nallar et al., 2010; Jensen et al., 2012). Enhanced sensory experience increases Wnt7a/b levels in the adult hippocampus, which act as retrograde signals inducing profound presynaptic plasticity-related modeling of CA3-CA1 mossy fiber synapses (Gogolla et al., 2009). Importantly, local application of the Wnt antagonist Sfrp1 by canulla injection during enhanced sensory experience prevents experience-dependent presynaptic remodeling and terminal complexity (Gogolla et al., 2009). Moreover, Wnt signaling acutely enhances the frequency and amplitude of spontaneous and evoked EPSCs in mature hippocampal neurons (Beaumont et al., 2007; Varela-Nallar et al., 2010; Ciani et al., 2011). These findings suggest that Wnts may contribute to activity-dependent plasticity. Indeed, a role for Wnt in LTP has been suggested. In acute hippocampal brain slices activation of the canonical-Wnt signaling by exogenous Wnt3a or lithium weakly facilitates LTP, whereas specific antibodies against Wnt3a or the secreted extracellular domain of Fz8 decreases LTP (Chen et al., 2006). More recently, Wnt5a has also been shown to facilitate LTP at CA3-CA1 synapses (Cerpa et al., 2011). These studies strongly suggest that Wnts modulate LTP. However, loss-of-function studies are required to fully demonstrate whether Wnt signaling is required for LTP.

### Wnt signaling in synaptic maintenance

*In vivo* imaging demonstrates that 70–90% of synapses in the mature brain are stable and persist for extended periods of time, perhaps for the lifespan of the animal (Trachtenberg et al., 2002; Meyer et al., 2003; Alvarez and Sabatini, 2007; Bhatt et al., 2009; Holtmaat and Svoboda, 2009). Synaptic function and maintenance do not only determine stimulus transmission in the brain, but also the stability of axonal and dendritic arbors (Wu and Cline, 1998; Rajan et al., 1999; Niell et al., 2004; Hu et al., 2005; Meyer and Smith, 2006; Ruthazer et al., 2006; Chen et al., 2010). During development, motile “exploratory” axonal branches form preferentially at nascent presynaptic sites; in contrast stable presynaptic sites inhibit dynamic axon behaviors and growth, and are associated with stable axonal branches (Meyer and Smith, 2006). These studies are supported by the finding of a direct correlation between synaptic maintenance and local axon stability, which is enhanced by neuronal activity (Ruthazer et al., 2006). The relationship between synaptic and axonal maintenance is equally reflected in dendritic arbor structure, where loss of synapses is associated with dendritic retraction (Sfakianos et al., 2007; Lin and Koleske, 2010). Together, the findings suggest that axon and dendrite stability and the preservation of neuronal networks are determined by synaptic maintenance.

In contrast to stable synaptic structure, synaptic proteins are highly labile with half-lives of just days or even less (Huh and Wenthold, 1999; Ehlers, 2003). Synaptic proteins shuttle in and out of synapses at a remarkable rate through lateral diffusion or exo- and endocytosis (Malinow and Malenka, 2002; Bredt and Nicoll, 2003; Wenthold et al., 2003; Collingridge et al., 2004; Cognet et al., 2006; Gray et al., 2006; Ehlers et al., 2007; Kielland et al., 2009). Synaptic proteins such as Ras, Shank, PSD-95, Bassoon and Synaptophysin are redistributed between adjacent synapses over a time scale of minutes and hours (Kim and Sheng, 2004; Gray et al., 2006; Tsuriel et al., 2006, 2009). Furthermore, highly motile SVs are recycled between multiple presynaptic sites (Darcy et al., 2006; Staras et al., 2010). These studies raise a fundamental question; if synaptic components are only transiently located to synaptic sites, how is the long-term integrity of the synapse maintained?

**Figure 4:**
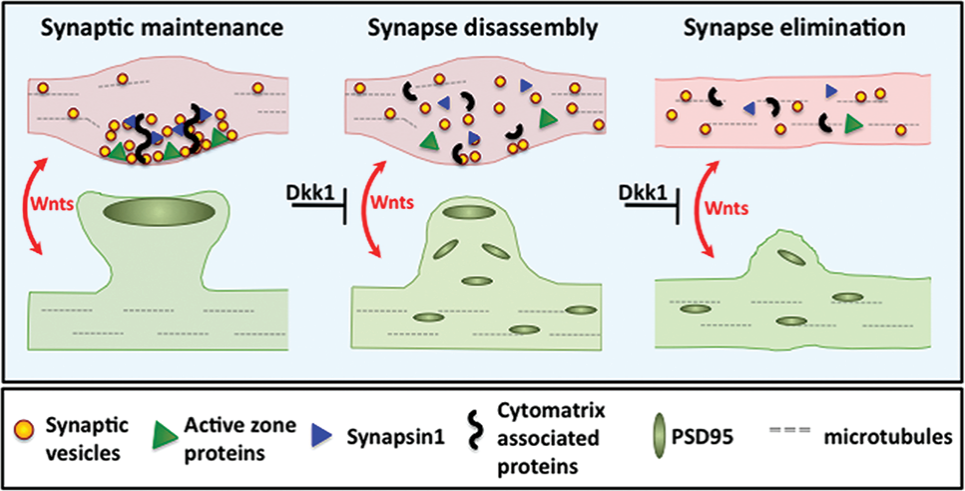
**Wnt blockade by Dkk1 induces synapse disassembly**. Left panel: Wnt signaling maintains pre- and postsynaptic stability. Middle panel: Blockade of Wnt signaling by Dkk1 induces dispersal of synaptic proteins. During Wnt blockade, functionally diverse synaptic components such as SVs, Synapsin1 and active zone proteins disperse from presynaptic sites. Postsynaptically, PSD95 also disperses. Right panel: Synapse disassembly eventually leads to synapse elimination.

Synaptogenic factors are ideally suited to modulate synaptic stability. Indeed, Wnt signaling has now been demonstrated to regulate synaptic maintenance in mature neurons. Time-lapse imaging experiments demonstrate that Wnt signaling blockade by the secreted antagonist Dkk1 eliminates previously stable presynaptic sites within 20 min (Purro et al., 2012). Dkk1 rapidly induces the delocalization of pre- and postsynaptic components in mature and stable hippocampal synapses (Figure 4). This effect is accompanied by a reduced number of SV recycling sites. Importantly, ultrastructural analyses of remaining synapses reveal smaller active zones and postsynaptic densities, suggesting coordinated pre- and postsynaptic shrinkage and disassembly by Wnt blockade with Dkk1. These synaptic changes occur in the absence of cell death or decreased cell viability (Purro et al., 2012), suggesting that Dkk1 directly affects the synapse. These findings provide evidence that endogenous Wnt signaling is required for synaptic maintenance.

Synaptic activity is the key stimulus for maintaining the molecular composition of synapses. Several synaptic proteins and signaling molecules are synthesized in an activity dependent manner (Steward and Schuman, 2001; West et al., 2001). Activity-dependent mechanisms also regulate the trafficking of synaptic proteins to active synaptic sites (Ehlers et al., 2007; Kielland et al., 2009). As discussed above, neuronal activity regulates the expression and release of Wnts. Moreover, Wnt blockade induces rapid synapse disassembly (Purro et al., 2012). We therefore propose that neuronal activity promotes the stability of synapses by modulating the levels of Wnts. Conversely, at inactive synapses limited levels of Wnts could render these synapses more prone to destabilization and disassembly.

Mounting evidence suggests that in several neurodegenerative diseases, the loss of synapses correlates well with cognitive decline and motor impairment before cell death is evident. These findings highlight the importance for understanding the cellular and molecular mechanisms that control the stability of synapses while permitting plastic changes to occur.

### Dysfunctional Wnt signaling in neurodegenerative disorders

Dysfunctional Wnt signaling is linked to a number of neurological disorders including autism, schizophrenia, bipolar disorders, Alzheimer’s disease (AD) and Parkinson’s disease (De Ferrari and Moon, 2006; Inestrosa and Arenas, 2010; Okerlund and Cheyette, 2011; Scott et al., 2013). A common feature underlying these neurological disorders is aberrant synapse function and/or synapse degeneration. Synapse disassembly often precedes and might even trigger the widespread and catastrophic cell death that hallmarks many neurodegenerative disorders (Selkoe, 2002; Luo and O’Leary, 2005; Saxena and Caroni, 2007; Shankar et al., 2007; Stevens et al., 2007; Rosen and Stevens, 2010; Kessels et al., 2013). Biopsies of human AD brains reveal a ∼15–35% loss of synapses per neuron within 2–4 years of clinical AD onset (Terry et al., 1991). Importantly, the degree of cognitive decline correlates with loss of Synaptophysin-labeled presynaptic sites (Masliah et al., 2001). These findings are consistent with the results obtained from transgenic mouse models of AD, where spine loss is best correlated with cognitive impairment than with the deposits of Amyloid plaques (Knobloch and Mansuy, 2008). Synapse loss in the AD brain correlates with the accumulation of soluble Amyloid-β (Aβ) prior to the onset of neuronal cell death, suggesting that soluble Aβ contributes to synaptic defects (Hsia et al., 1999; Selkoe, 2002; Lacor et al., 2007; Shankar et al., 2007). Indeed, Aß modulates synaptic molecules resulting in synaptic dysfunction (Cisse et al., 2011). However, the molecular mechanisms by which soluble Aβ promotes synapse loss are poorly understood. Together the findings strongly suggest that synapse disassembly is an early event in AD.

A potential role for Wnt signaling in the pathogenesis of AD has been suggested by a number of recent studies (De Ferrari and Moon, 2006; Caraci et al., 2008; Inestrosa and Arenas, 2010; Purro et al., 2012). Exogenous Wnt5a ameliorates Aβ-induced cell death in hippocampal neurons (De Ferrari et al., 2003; Cerpa et al., 2010). In addition, Wnt3a rescues Aβ-mediated loss of neurogenesis and neural differentiation in embryonic hippocampal progenitor cells *in vitro* (Shruster et al., 2011). Soluble Aβ binds to Fz5 and blocks canonical Wnt signaling in NB2A cells (Magdesian et al., 2008). Increased Gsk3β activity, which inhibits Wnt signaling, induces the hyperphosphorylation of Tau and adversely affects Amyloid precursor protein (APP) processing (Mudher et al., 2001; Hooper et al., 2008). However, the first clear link between Wnt signaling and AD came from a genome-wide association study demonstrating that a genetic variant of LRP6, the receptor for Dkk1, is strongly associated with late-onset AD (De Ferrari et al., 2007). This variant of LRP6 exhibits decreased level of Wnt signaling. Together these results suggest that decreased levels of Wnt signaling could contribute to Aβ-mediated neuropathogenesis.

**Figure 5:**
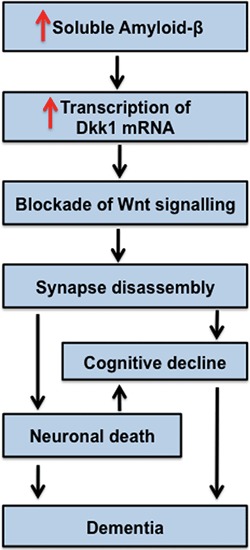
**Model for Dkk1 in the progression of AD.** Increased levels of soluble Amyloid-β stimulate the transcription of Dkk1, which blocks endogenous Wnts important to the stability of synapses. Thus, Dkk1 induces synapse disassembly by the dispersal of synaptic components. Loss of synapses correlates with cognitive decline. We propose that Dkk1-induced synapse elimination is an early event in the progression of AD. The notion that synapse disassembly precedes cell death is in accordance with current models for the progression of AD.

Further links between dysfunction of Wnt signaling and AD comes from studies on the Wnt antagonist Dkk1. Dkk1 expression is elevated in human postmortem AD brains and in mouse models of AD (Caricasole et al., 2004; Rosi et al., 2010). In hippocampal brain slices, acute exposure to Aβ oligomers rapidly increases Dkk1 mRNA and Dkk1 protein levels in the CA1, CA3 and DG (within 3 hrs), with a concomitant loss of synapses (Purro et al., 2012). Crucially, blockade of Dkk1 with specific neutralizing antibodies suppresses the ability of Aβ to stimulate synapse loss (Purro et al., 2012). These studies demonstrate that Dkk1 is required for Aβ-induced synapse elimination. We propose that dysfunction in Wnt signaling contributes to Aß-mediated synaptic toxicity and the subsequence cognitive decline observed in dementia (Figure 5).

How does Aβ induce Dkk1 expression? A recent report suggests that increased soluble Aβ induces the intracellular accumulation of the cell survival factor clusterin, possibly by preventing its neural secretion (Killick et al., 2012). It was posited that intracellular clusterin accumulation activates p53 signaling, which stimulates Dkk1 expression. Consistent with this idea, knockdown of clusterin prevents Aβ toxicity and upregulation of Dkk1 in cultured hippocampal neurons. Moreover, pharmacological inhibition of the p53 pathway blocks Aβ induction of Dkk1 (Killick et al., 2012). Thus, p53 activation contributes to Aβ-mediated Dkk1 induction. It would be critical to establish whether p53 also contributes to Dkk1 increased levels during synaptic disassembly induced by Aβ *in vivo*.

Recent studies have shown that endogenous Dkk1 expression becomes progressively elevated in the aged brain and may be associated with general cognitive decline as well as susceptibility to age related pathological neurodegenerative disorder (Scott et al., 2013; Seib et al., 2013). Indeed, Dkk1 knockdown increases neurogenesis in the adult hippocampus and this effect has been correlated with enhanced working spatial memory and memory consolidation (Seib et al., 2013). However, it remains to be examined whether changes in spatial memory are due to changes in synapse integrity.

## Concluding remarks

The delicate balance between synapse assembly, disassembly and maintenance are highly regulated processes controlled by a complex molecular dialogue between pre- and postsynaptic neurons. Importantly, neuronal activity, which has profound effects on these processes, contributes to the expression and release of Wnt factors, which in turn modulate this trans-synaptic dialogue. Indeed, several studies have demonstrated that Wnts contribute to activity-mediated synapse formation during early neuronal circuit development and during experience-dependent plasticity.

Wnt-mediated synaptic maintenance is now emerging in relation to neurodegeneration. Indeed, the Wnt antagonist Dkk1, which has strong synaptic disassembly activity, is now linked to AD. Importantly, Dkk1 mediates the effect of Aβ on synaptic disassembly. Future *in vivo* studies will provide key evidence for the contribution of dysfunction in Wnt signaling to synaptic degeneration in AD.

It is an exciting time to study Wnt signaling in the nervous system as we begin to unravel the mechanisms by which Wnts modulate experience-mediated plasticity. Moreover, the potential role of Wnts in synaptic maintenance opens new avenues to develop therapeutic strategies for the treatment of neurodegenerative diseases at early stages. Finally, the finding that Dkk1 is elevated in the AD brain has sparked enthusiasm that this molecule could be used as a biomarker at early stages of AD or other neurodegenerative diseases.

## Conflict of interest statement

The authors declare that the research was conducted in the absence of any commercial or financial relationships that could be construed as a potential conflict of interest.
